# Supplementary material for: Patterns of Intron Gain and Loss in Fungi
Source: PLoS Biol. 2004 Nov 30;2(12):e422. doi: 10.1371/journal.pbio.0020422 (PMC532390; doi:10.1371/journal.pbio.0020422)
Supplement: Table S1 — Also available at http://genes.mit.edu/NielsenEtAl/. (4.3 MB ZIP). [file pbio.0020422.st001.zip › NielsenEtAl/html/744.html]

AN0250.1.NCU06138.1.MG05946.1.FG09052.1


```
 CLUSTAL W (1.82) Multiple Sequence Alignments - Introns Inserted


Sequence 1: MG05946.1	578 aa
Sequence 2: FG09052.1	557 aa
Sequence 3: NCU06138.1	583 aa
Sequence 4: AN0250.1	547 aa
Alignment Length: 588 aa
Number Identitical Residues: 283 aa
Alignment Score (without introns) 14394


MG05946.1 	MP--AAAGDSGGYGLALERREAMMGAS-GPRAILKNFKVFRVALFACIGG~ILYGYNQ~G
NCU06138.1	MGGGGGSGAAGFYDAALQRREAVMGKS-GPAALVKNFRVFSIACFACIGG~VLYGYNQ~G
FG09052.1 	MG--GAPTTVGTYDAALQRRQALMGAS-GARALVKNWKVFRIAAFACIGG~ILYGYNQ~G
AN0250.1  	MG--GAGDTSAALDAAAQIRKVNLKASSGLPGLIQNKKVFGVAMFACLGG2LLYGYNQ0G
          	*   ..    .  . * : *:. :  *:*  .:::* :** :* ***:** :****** *

MG05946.1 	MFSGVL~AMPSFQRH1MGEWAPLDPEADQAKKGWLTA~ILELGAWIGTLLSSFIAEILSR
NCU06138.1	MFSGVL~AMPAFQKH1MGEYDPIDENASQTKKGWLTA~ILELGAWLGTLLSGFMAEVLSR
FG09052.1 	MFSGIL~VMKSFERH1MGDYV-----QNKTKKGWLTS~ILELGAWLGTLMSGAIAELCSR
AN0250.1  	VFSGVL1VMHSFEEH0MGDAV-----TNETKKGWLTS1ILELGAWLGALYSGVLCERFSR
          	:***:* .* :*:.* **:        .::******: *******:*:* *. :.*  **

MG05946.1 	KYGILVACAVFMIGVVIQACAVTIDVDLAHNAILAGRFIT2GMGVGS~LAMIIPIYNSE~
NCU06138.1	KYGVLVACLVFMLGVVIQATSISGG----HETILAGRFIT2GMGVGS~LAMIIPIYNSE~
FG09052.1 	KYGILIATSVFIIGVVIQCTAMQAG----HNAILAGRFIT2GMGVGS~LSTIVPLYNSE~
AN0250.1  	KYTILAHVCIFCIGVVIQLTAAAGSG--SSSNILGGRFVT~GMGVGS2LSMSVPMYNAE0
          	** :*    :* :*****  :   .   : . **.***:* ****** *:  :*:**:* 

MG05946.1 	VAPP~E~VRGALVALQQLSICFGIMISFW~IDY2GTNFIGGTTVETQLDAAWLTPTTLQL
NCU06138.1	VAPP~E~VRGALVALQQLAICFGIMVSFW~IDY2GTNYIGGTKLETQSDAAWLVPVCLQL
FG09052.1 	CAPP2E~VRGALVALQQLAITFGIMVSFW~IDY2GTHFIGG-TGEGQSDAAWLIPVCLQL
AN0250.1  	IAPP~E0VS------------------FW2INY~GTNHIGG-TGETQKDTAWLLPLALQL
          	 *** * *                   ** *:* **:.*** . * * *:*** *  ***

MG05946.1 	FPALVLFIGMIFMPFSPRWLIHHDREEDGIRVLANLRGLPSDNELIQLEFLEIKAQSLFE
NCU06138.1	APALILFFGMMFMPFSPRWLIHHGREAEARKILSTLRGLPQDHELVELEFLEIKAQSLFE
FG09052.1 	GPAIILFIGIMWMPFSPRWLTHHNREEEARQVLANLRDLPTDHELIELEFLEIKAQSLFE
AN0250.1  	VPAVILGVGMIFMPFSPRWLVHHGREEEALKVLTSLRGLEADDPVLQLEFLEIKAQSVFE
          	 **::* .*:::******** **.** :. ::*:.**.*  *. :::**********:**

MG05946.1 	KRSVAEQFPQLSNPTAWNTFKLQFVAIGRLFQTRSMWKRIIVATV~TMFFQQWTGINAVL
NCU06138.1	KRSIAELFPELREQTAWNTFKLQFVAIEKLFRTKAMFRRVIVATV~TMFFQQWSGINAIL
FG09052.1 	KRSLAEKFPHLQEQTAGNTIKLQFVAIAALFKTKAMFKRVIVATV~TMFFQQWTGIN---
AN0250.1  	KRTEKERFPHLERSSLWSYLKLEAAGYKSLFTSWPMFKRVIVATV1TMTFQQWTGVNAIL
          	**:  * **.* . :  . :**: ..   ** : .*::*:***** ** ****:*:*:  

MG05946.1 	YYAPS~IFKQLGLSNNEISLLATGVVGVVM0FIAT~IPAVLWIDRAGRKPVLTIGALGMS
NCU06138.1	YYAPQ~IFKQLGLSGNTTSLLATGVVGIVM~FIAT~VPAVLWIDRVGRKPVLTIGALGMA
FG09052.1 	----A0IFEQLGLSSNTTSLLATGVVGIVM~FIAT~IPAVLWIDRLGRKPVLAVGAIGMG
AN0250.1  	YYAPT~IFEQLGMSSNTTSLLATGVVGVAM~FLAT0IPAVIWVDNVGRKPILLIGAIGMA
          	  :.  **:***:*.*  *********:.* *:** :***:*:*. ****:* :**:**.

MG05946.1 	FCHFV2IAIILAKNIDRFDEQKAAGWAACAMVWLFVIHFGY~SWGPCAWIIIAEIWPMSV
NCU06138.1	TCHII~IAVIVAKNVDQWETHKAAGWAAVAMVWLFVIHFGY~SWGPCAWIIVAEIWPLST
FG09052.1 	LCHLI~IAVILARNIDQFDTHPAAGWAAICMVWLFVVHFGY1SWGPCAWIIIAEIWPLST
AN0250.1  	ACHFI~IAGIFAENETQWESHRAAGWGAVAMVWLFVIHFGY~SWGPCSWIIIAEIWPLSV
          	 **:: ** *.*.*  ::: : ****.* .******:**** *****:***:*****:*.

MG05946.1 	RPYGVALGASSN~WM2NNFIVGQVTPIMLQKITYGTYLLFAILTLMGACFIYFFVPETKR
NCU06138.1	RPYGVSLGASSN~WM~NNFIVGQVTPDMLKAIPYGTYIIFGLLTYMGAAFIWFFVPETKR
FG09052.1 	RPYGVALGASSN~WM2NNFIVGQVTPEMLDGITYGTYILFGLLTMIGAAFIWFFVPETKR
AN0250.1  	RAIGTSLGASAN1WM~NNFIVGQVTPDMLAGIRYGTYIFLGLMTTLGALFIFFFVPETKQ
          	*. *.:****:* ** ********** **  * ****:::.::* :** **:*******:

MG05946.1 	LTLEEM0DLVFGSSGTTQADLERQREINIEVGLDRAAG-ALGTSERPHSLSD---EKEKQ
NCU06138.1	LTLEEM0DMIFGSEGTAQADNERMEEINAEIGLTRFLQGGSGANQGAADGSDTGYDAEKG
FG09052.1 	LSLEEM0DIIFGSEGAAQADNERMAEINREIGLDDLIY-RSGAVHSNEDIHQ---DVKDV
AN0250.1  	VSLEEM~DVIFGSEGTAVADHERQAEISREIGLDEALA--RFSTRSGATLAE----VEPK
          	::**** *::***.*:: ** **  **. *:**         : .      :     :  

MG05946.1 	KTDAVV---
NCU06138.1	KSEHYSQHV
FG09052.1 	KE-------
AN0250.1  	NA-------
          	:
```
